# Putting patients at the centre of eye care

**Published:** 2012

**Authors:** Sally Crook

**Affiliations:** Programme manager: Seeing is Believing and Consulting Editor for this issue.

**Figure:**
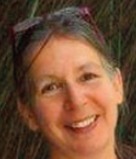
Sally Crook

Managing a successful eye clinic can be very challenging. We may have many questions:

- How can we make the most efficient use of our staff and resources?
- Is it possible to provide a better service without increasing our costs?
- Can we increase the number of patients we serve?
- Are our patients satisfied with what we offer?

The last question is probably the most important one we can ask ourselves. Satisfied patients talk to others in the community, promote our clinic to others, and encourage those who are fearful to come for treatment.

Higher patient numbers can provide us with additional income and is evidence that we are delivering much-needed services. All of this will help to ensure our success.

Keeping patients in mind can also help us to improve. If we want to provide a better and more efficient service, while keeping costs low, it can be very confusing to know where to start. There is so much to think about: costs, facilities, equipment, planning, record-keeping, and so on. But if we focus on how to improve patients' experiences and how to make them more satisfied, we will be well on our way. Even a tiny change – ensuring the toilets in the waiting area are clean and tidy, for example – could affect how patients feel and what they say to others.

Staff also benefit if the clinic puts patients at the centre of what they do. Treating staff with respect, and rewarding them for providing patient-centred care, creates a positive and caring environment for everyone, including patients!

In this issue, we will show you how to find out what patients really think about your clinic, how to better organise your services with your patients in mind, and how to reach beyond the clinic to encourage more patients to come forward. We have also included several case studies illustrating how patient-centred eye care services can have a better impact on the whole community.

**Figure:**
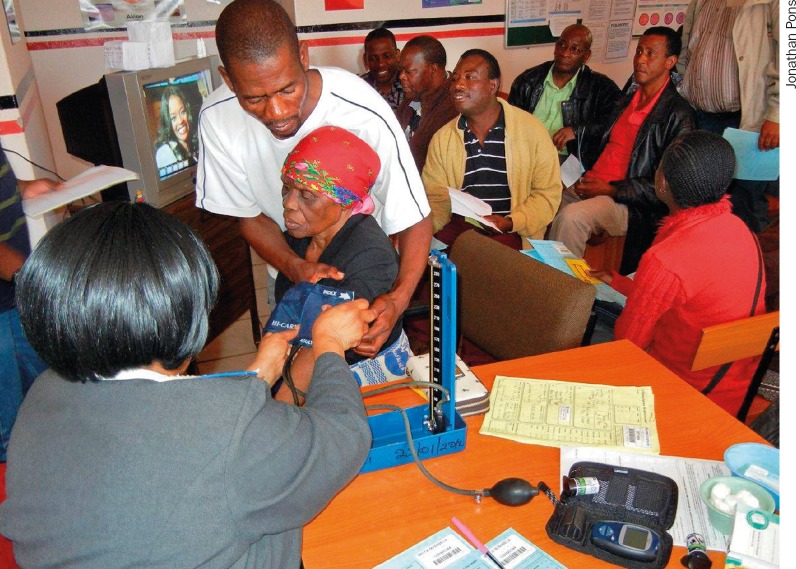
With patients at the centre of what we do, everyone benefits. SWAZILAND

We hope that this issue will give you the inspiration and tools you need to make a difference where you work.

